# Non-albumin proteinuria as a candidate biomarker of treatment response to finerenone in diabetic kidney disease: a real-world cohort study

**DOI:** 10.3389/fendo.2026.1918192

**Published:** 2026-09-08

**Authors:** Min Seo Choi, Hyung Woo Kim, Minheui Yu, Minyoung Lee, Yong-ho Lee, Eun Seok Kang, Bong-Soo Cha, Byung-Wan Lee

**Affiliations:** 1 Department of Internal Medicine, Yonsei University College of Medicine, Seoul, Republic of Korea; 2 Department of Internal Medicine, Institute of Kidney Disease Research, Yonsei University College of Medicine, Seoul, Republic of Korea; 3 SENTINEL Team, Division of Endocrinology, Department of Internal Medicine, Yonsei University College of Medicine, Seoul, Republic of Korea

**Keywords:** albuminuria, diabetic nephropathies, finerenone, personalized medicine, proteinuria, real-world evidence, tubular injury, type 2 diabetes

## Abstract

**Introduction:**

Finerenone reduces albuminuria and improves kidney outcomes in patients with diabetic kidney disease (DKD); however, its association with changes in non-albumin proteinuria (NAP), enriched for tubular injury-related proteins, remains unclear. We evaluated whether finerenone treatment was associated with a reduction in NAP and identified clinical phenotypes associated with a greater NAP response.

**Methods:**

We retrospectively analyzed 477 patients treated with finerenone who had baseline urine albumin-to-creatinine ratio (uACR) and urine protein-to-creatinine ratio (uPCR) measurements from the same urine specimen. Urine non-albumin protein-to-creatinine ratio (uNAPCR) was calculated as uPCR minus uACR. Patients were stratified by baseline proteinuria severity (uPCR < 500 vs ≥ 500 mg/gCr). The primary outcome was the geometric mean percentage change in uNAPCR after 6 months of finerenone treatment, assessed using linear mixed-effects models. Subgroup interaction analyses were performed to assess differential associations and identify factors associated with greater uNAPCR reduction.

**Results:**

At baseline, median uPCR, uACR, and uNAPCR were 1140 [580, 2290], 716 [328, 1593], and 410 [223, 700] mg/gCr, respectively. At 6 months, uACR and uNAPCR declined by 30.3% and 17.5%, respectively. Subgroup analyses revealed differential associations: patients with higher baseline proteinuria (uPCR ≥ 500 mg/gCr) showed an 18.8% decline in uNAPCR, whereas those with uPCR < 500 mg/gCr showed a non-significant 1.3% change. Changes in uNAPCR were positively correlated with changes in urine N-acetyl-β-D-glucosaminidase-to-creatinine ratio (uNAGCR) (Spearman’s ρ = 0.53; p < 0.001).

**Discussion:**

Finerenone treatment was associated with a reduction in NAP, particularly among patients with higher baseline proteinuria, suggesting a possible effect on tubulointerstitial injury-related processes beyond albuminuria reduction. Prospective multicenter studies are needed to validate uNAPCR as a treatment-response biomarker against clinical endpoints.

## Introduction

Diabetic kidney disease (DKD) is a common and clinically important microvascular complication of diabetes (1). Albuminuria has conventionally been used as an early marker of DKD, and a decline in estimated glomerular filtration rate (eGFR) reflects progression (2). However, the classical paradigm that albuminuria necessarily precedes eGFR decline in DKD does not hold uniformly across patients. In particular, patients with DKD who exhibit a decline in kidney function without accompanying increases in albuminuria highlight the heterogeneity of DKD (3). This heterogeneity exposes a limitation of the current albuminuria-centric paradigm for DKD risk stratification and treatment selection, and highlights the need for complementary biomarkers that can support more personalized management.

In this context, non-albumin proteinuria (NAP) may provide complementary information for characterizing DKD phenotype. Because it comprises non-albumin proteins, including low-molecular-weight proteins associated with tubular injury, NAP may carry information on the tubulointerstitial compartment that albuminuria, a predominantly glomerular signal, does not fully capture (4). Consistent with this, higher NAP has been associated with chronic kidney disease (CKD) progression and with mortality in type 2 diabetes (T2D) independently of albuminuria, and it can be elevated even when albuminuria is absent (5–7).

Finerenone is a nonsteroidal, highly selective mineralocorticoid receptor (MR) antagonist with a distinct binding mode that reduces MR cofactor recruitment (8–10) and modulates pro-inflammatory and pro-fibrotic gene expression (11–13). MR overactivation in diabetes promotes oxidative stress, inflammation, and fibrosis not only within the glomerulus but along the tubulointerstitial axis (14), and in preclinical models finerenone attenuates tubular inflammation and interstitial fibrosis (15, 16). The pivotal Finerenone in Reducing Kidney Failure and Disease Progression in Diabetic Kidney Disease (FIDELIO-DKD) and Finerenone in Reducing Cardiovascular Mortality and Morbidity in Diabetic Kidney Disease (FIGARO-DKD) trials evaluated finerenone in patients with T2D and CKD. These trials and their prespecified pooled analysis, Finerenone in Chronic Kidney Disease and Type 2 Diabetes: Combined FIDELIO-DKD and FIGARO-DKD Trial Programme Analysis (FIDELITY), showed that finerenone reduced albuminuria and lowered the risk of cardiorenal events (17–19). Yet this entire evidence base is anchored to albuminuria; data on whether finerenone’s anti-inflammatory and anti-fibrotic actions extend to the non-albumin, tubular-enriched fraction of proteinuria remain limited.

We therefore hypothesized that the anti-inflammatory and anti-fibrotic effects of finerenone extend beyond the glomerular, albumin-dominated compartment, and that finerenone treatment would accordingly be accompanied by a quantifiable reduction in NAP. To test this hypothesis, we evaluated changes in NAP levels before and after finerenone treatment and examined clinical parameters associated with a greater decline in NAP. By characterizing how baseline clinical phenotype relates to the change in NAP after finerenone initiation, we sought to evaluate the urine non-albumin protein-to-creatinine ratio (uNAPCR) as a candidate biomarker of treatment response that could inform a more personalized approach to the management of DKD.

## Materials and methods

### Study design

This single-center, real-world, retrospective cohort study used electronic medical records (EMR) from Severance Hospital. Patients with T2D who received finerenone between February 20, 2024, and April 30, 2025, were screened. Eligible patients were required to meet all of the following criteria (1): age ≥20 years with a confirmed diagnosis of T2D (2); finerenone prescription for ≥90 cumulative days; (3) availability of both baseline urine albumin-to-creatinine ratio (uACR) and urine protein-to-creatinine ratio (uPCR) measured from the same urine specimen before finerenone initiation. We excluded patients with a baseline uACR ≥5,000 mg/gCr and those treated with immunosuppressive or antineoplastic agents before or during finerenone treatment. Hypertension was not applied as an inclusion criterion, so that patients without documented hypertension who exhibited the DKD phenotype of interest were retained; all included patients were nonetheless receiving an angiotensin-converting enzyme inhibitor (ACEi) or angiotensin receptor blocker (ARB) at baseline. Hypertension was instead accounted for analytically, as a prespecified adjustment covariate in all linear mixed-effects models and as a prespecified subgroup in the interaction analysis. The study protocol was approved by the Institutional Review Board of Severance Hospital (approval no. 4-2025-0126), and the requirement for informed consent was waived owing to the retrospective design.

### Exposure, measurements and definitions

The index date was the first finerenone prescription; analyses were restricted to the first continuous treatment episode (consecutive fills separated by gaps ≤45 days). Adherence was quantified as the proportion of days covered (PDC); high adherence (PDC ≥80%) was used in sensitivity analyses. Baseline demographics, comorbidities, medications, and laboratory data were extracted from the EMR. eGFR was calculated using the 2009 Chronic Kidney Disease Epidemiology Collaboration (CKD-EPI) equation (20). All urinary measurements were performed in the central clinical laboratory of Severance Hospital using the same analytical platform throughout the study period. Urine non-albumin protein-to-creatinine ratio (uNAPCR)—a measure of NAP—was calculated as uPCR minus uACR, using values obtained from the same urine specimen [uNAPCR (mg/gCr) = uPCR (g/gCr) × 1,000 − uACR (mg/gCr)]. Urine albumin was measured using an immunoturbidimetric method, whereas total urine protein was measured by a colorimetric dye-binding method with different calibration and analyte specificity. Urine N-acetyl-β-D-glucosaminidase-to-creatinine ratio (uNAGCR) was calculated as 100 × uNAG/urine creatinine.

### Outcomes

The primary outcome was the geometric mean percentage change in uNAPCR from baseline to 6 months. Secondary outcomes were geometric mean percentage changes in uACR and uPCR at 6 months. As an exploratory mechanistic analysis, we examined the association between the change in uNAPCR and the change in uNAGCR, a well-established urinary marker of tubular injury. Safety outcomes were hyperkalemia (serum potassium >5.5 mmol/L) and severe hyperkalemia (>6.5 mmol/L).

### Statistical analysis

Continuous variables are presented as mean (SD) or median (interquartile range, IQR) as appropriate, and categorical variables as number (%). Urinary protein measures (uPCR, uACR, and uNAPCR) were log-transformed before analysis because of their non-normal distribution; geometric means and percentage changes were obtained by back-transformation. Changes from baseline to 6 months were estimated using linear mixed-effects models with patient-level random intercepts and random slopes for time, adjusted for age, sex, body mass index (BMI), hypertension, hemoglobin A1c (HbA1c), baseline sodium-glucose cotransporter 2 inhibitor (SGLT2i) use, baseline eGFR, and baseline proteinuria category (uPCR <500 vs ≥500 mg/gCr); estimates were exponentiated and reported as geometric least-squares mean percentage changes with 95% confidence intervals (CIs). Differential responses were assessed with prespecified time × subgroup interaction terms. Baseline proteinuria severity (uPCR <500 vs ≥500 mg/gCr) was the prespecified primary stratifier; additional prespecified subgroups were defined by age, sex, BMI, hypertension, HbA1c, eGFR, and baseline SGLT2i use. This threshold was selected *a priori* because Kidney Disease: Improving Global Outcomes (KDIGO) 2012 places uPCR >500 mg/gCr in the range approximately corresponding to A3 albuminuria (21), and KDIGO 2024 identifies uPCR ≥500 mg/gCr as approximately equivalent to uACR ≥300 mg/gCr in its specialist kidney care referral criteria (22). Baseline uNAPCR tertiles were additionally analyzed as an independent stratification of NAP burden.

For the mechanistic analysis, within-patient changes in uNAGCR and uNAPCR (before vs after finerenone) were related using Spearman’s rank correlation (ρ), partial rank correlation, and multivariable linear regression, the latter two adjusted for the same covariates plus baseline uNAPCR. As a sensitivity analysis, the rank correlation was repeated after entropy-balancing calibration weighting, which reweighted the association subset to the covariate means of the full analytic cohort with respect to age, sex, BMI, HbA1c, hypertension, baseline SGLT2 inhibitor use, eGFR, and log-transformed baseline uNAPCR. Balance was assessed using absolute standardized mean differences, and weight dispersion using the effective sample size. The weighted 95% CI was obtained by patient-level bootstrapping and the two-sided p value by permutation. Safety analyses included all finerenone recipients regardless of the ≥90-day criterion and summarized hyperkalemia overall and by baseline eGFR stratum. Robustness was examined in prespecified sensitivity analyses — the high-adherence subgroup (PDC ≥80%) and a pre- versus post-index piecewise-slope model. Two-sided p < 0.05 denoted statistical significance; all analyses used R 4.3.3 (R Foundation for Statistical Computing, Vienna, Austria).

## Results

### Patients’ baseline characteristics

We retrospectively identified 834 patients with any finerenone prescription within the study window. After applying inclusion and exclusion criteria, 477 patients comprised the final analytic cohort (**Supplementary Figure 1**). Exclusions included <90 cumulative days of exposure during the first treatment episode (n=72), lack of paired same-specimen uACR and uPCR measurements (n=224), baseline uACR ≥5,000 mg/gCr (n=20), and use of immunosuppressive or antineoplastic agents (n=41). The study included 477 patients with a mean age of 66.3 ± 12.4 years. Of the participants, 71.3% were male. The median BMI was 25.7 kg/m² (IQR 23.5–28.3). All included patients had T2D and were receiving an ACEi or ARB at baseline, and 44.2% of the participants were on SGLT2i.

The mean HbA1c was 7.4 ± 1.4%. Baseline serum potassium concentration averaged 4.6 ± 0.4 mmol/L. The median eGFR was 53.7 mL/min/1.73 m²; 39.6% of participants were classified as G1-G2 (eGFR ≥60), 27.5% as G3a (45 to <60), and 32.9% as G3b-G4 (eGFR <45 mL/min/1.73 m²). Median uPCR, uACR, and uNAPCR were 1140, 716, and 410 mg/gCr, respectively. According to KDIGO albuminuria categories, 9 (1.9%), 93 (19.5%), and 375 (78.6%) participants were classified as A1, A2, and A3, respectively (**Table 1**). Except for urinary protein excretion and hypertension, no baseline characteristics differed significantly between subgroups stratified by proteinuria severity (uPCR ≥ 500 vs < 500 mg/gCr).

**Table 1:** Baseline demographic and clinical characteristics of the overall study cohort.

Characteristic	Total (n = 477)	uPCR < 500 mg/gCr (n = 95)	uPCR ≥ 500 mg/gCr (n = 382)	P value
Age (years)	66.3 (12.4)	66.9 (13.0)	66.2 (12.2)	0.46
Male, n (%)	340 (71.3)	64 (67.4)	276 (72.3)	0.35
Treatment duration (months)	8.0 [5.7, 11.2]	7.1 [4.0, 10.6]	8.2 [5.8, 11.3]	0.14
BMI (kg/m^2^)	25.7 [23.5, 28.3]	25.5 [23.3, 27.2]	25.8 [23.5, 28.4]	0.16
HbA1c (%)	7.4 (1.4)	7.3 (1.3)	7.4 (1.4)	0.54
Hypertension, n (%)	401 (84.1)	72 (75.8)	329 (86.1)	**<0.05**
LDL cholesterol (mg/dL)	75 (38)	68 (26)	76 (40)	0.23
Serum potassium (mmol/L)	4.6 (0.4)	4.6 (0.4)	4.6 (0.5)	0.54
eGFR (mL/min/1.73 m^2^) ¶	53.7 [41.0, 72.7]	55.0 [45.4, 68.2]	53.6 [39.0, 73.4]	0.23
GFR category‡, n (%)				
G1 (≥90)	46 (9.6)	9 (9.5)	37 (9.7)	
G2 (60 to <90)	143 (30.0)	30 (31.6)	113 (29.6)	
G3a (45 to <60)	131 (27.5)	33 (34.7)	98 (25.7)	
G3b (30 to <45)	121 (25.4)	21 (22.1)	100 (26.2)	
G4 (15 to <30)	36 (7.5)	2 (2.1)	34 (8.9)	
G5 (<15)	0 (0.0)	0	0	
uPCR (mg/gCr)	1140 [580, 2290]	300 [220, 425]	1510 [863, 2528]	**<0.001**
uPCR category, n (%)				
<150	8 (1.7)	8 (8.4)	0	
150 to <500	87 (18.2)	87 (91.6)	0	
≥500	382 (80.1)	0	382 (100)	
uACR (mg/gCr)	716 [328, 1593]	117 [57, 226]	1015 [525, 1790]	**<0.001**
Albuminuria category‡, n (%)				
A1 (<30)	9 (1.9)	9 (9.5)	0	
A2 (30 to <300)	93 (19.5)	81 (85.3)	12 (3.1)	
A3 (≥300)	375 (78.6)	5 (5.3)	370 (96.9)	
300 to <1,000	182 (38.2)	5 (5.3)	177 (46.3)	
≥1,000	193 (40.5)	0	193 (50.5)	
uNAPCR (mg/gCr)	410 [223, 700]	162 [121, 196]	484 [323, 801]	**<0.001**
Smoking^†^, n (%)				0.23
Never-smoker	270 (65.7)	55 (68.8)	215 (65.0)	
Ex-smoker	71 (17.3)	17 (21.3)	54 (16.3)	
Current-smoker	70 (17.0)	8 (10.0)	62 (18.7)	
Baseline drug, n (%)				
SGLT2i use	211 (44.2)	44 (46.3)	167 (43.7)	0.65
ACEi or ARB	477 (100)	95 (100)	382 (100)	

Data are expressed as mean (SD) for normally distributed continuous variables, median (interquartile range) for non-normally distributed continuous variables, and number (%) for categorical variables.

¶ The estimated glomerular filtration rate (eGFR) was calculated with the use of the Chronic Kidney Disease Epidemiology Collaboration equation.

† Smoking status was available for n = 411.

‡ eGFR and albuminuria categories were defined according to Kidney Disease: Improving Global Outcomes (KDIGO): G1, eGFR ≥90; G2, 60 to <90; G3a, 45 to <60; G3b, 30 to <45; G4, 15 to <30; G5, <15 mL/min/1.73 m²; A1, uACR <30; A2, 30 to <300; and A3, ≥300 mg/gCr. A3 was further subdivided into 300 to <1,000 and ≥1,000 mg/gCr.

ACEi, angiotensin-converting enzyme inhibitor; ARB, angiotensin receptor blocker; BMI, body mass index; HbA1c, hemoglobin A1c; LDL-C, low-density lipoprotein cholesterol; SGLT2i, sodium-glucose cotransporter 2 inhibitor; eGFR, estimated glomerular filtration rate; uPCR, urine protein-to-creatinine ratio; uACR, urine albumin-to-creatinine ratio; uNAPCR, urine non-albumin protein-to-creatinine ratio.Bold values indicate P < 0.05.

### Changes in urinary biomarkers

**Figure 1:**
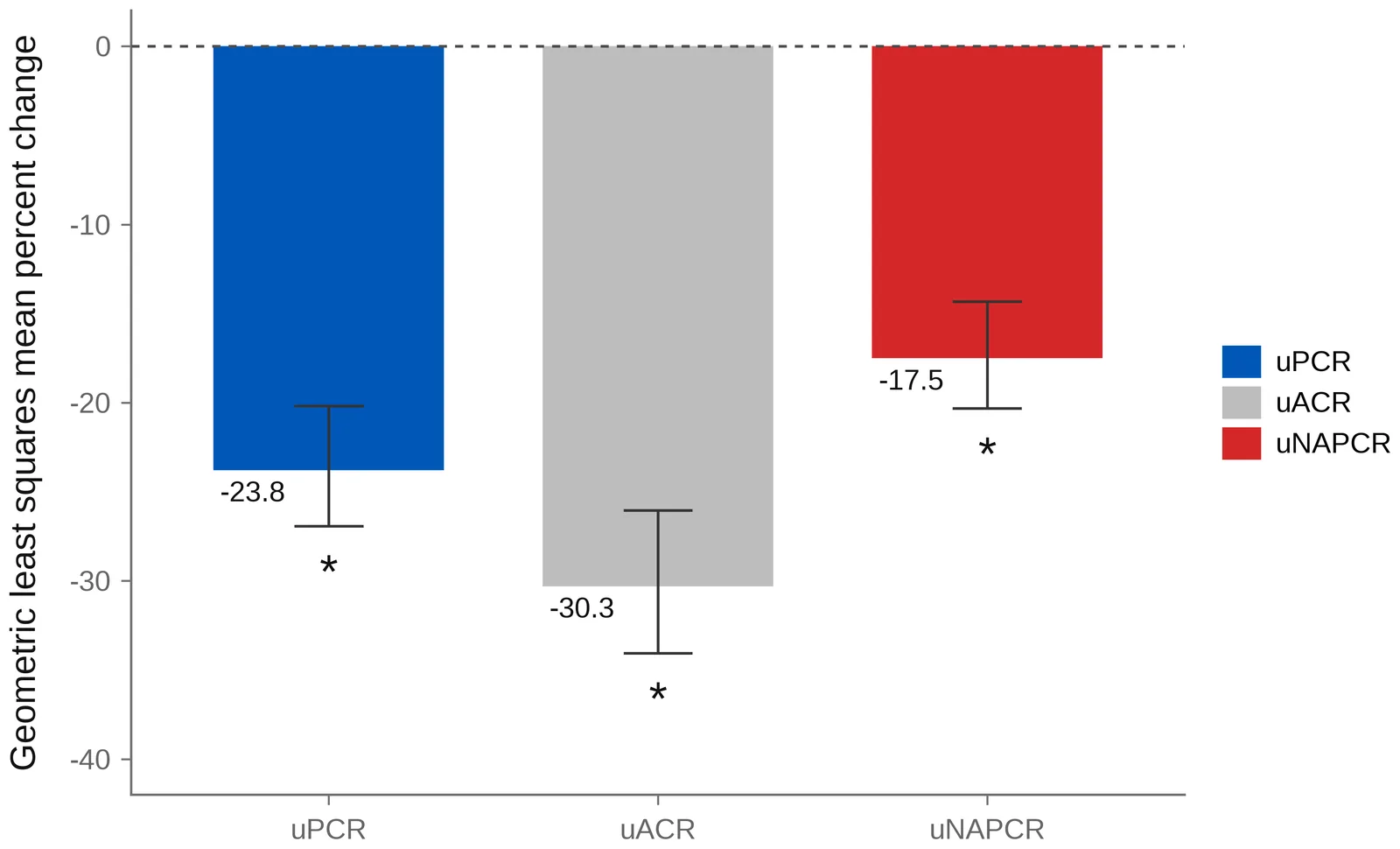
Geometric mean percentage changes in uPCR, uACR, and uNAPCR after 6 months of finerenone treatment. A linear mixed-effects model was used to estimate percentage change from baseline to 6 months, adjusting for age, sex, BMI, hypertension, HbA1c, SGLT2i use, eGFR and severity of proteinuria. The error bars denote 95% confidence intervals. *p < 0.05. uACR, urine albumin-to-creatinine ratio; uNAPCR, urine non-albumin protein-to-creatinine ratio; uPCR, urine protein-to-creatinine ratio.

**Figure 1** presents the geometric least-squares mean percentage changes in uPCR, uACR, and uNAPCR from baseline to 6 months of finerenone treatment, estimated using a linear mixed-effects model adjusted for baseline clinical covariates. From baseline to 6 months, uNAPCR declined significantly, with a change of −17.5% (95% CI, −20.6 to −14.4). Consistent with the pattern observed for uNAPCR, uPCR and uACR also showed significant decreases of −23.8% (95% CI, −27.1 to −20.3) and −30.3% (95% CI, −34.2 to −26.1), respectively. Among the 477 patients included in the analysis, 396 (83.0%) were classified as having high adherence, defined as PDC ≥ 80%. In this high-adherence subgroup, the estimated 6-month reductions in uNAPCR, uPCR, and uACR (−17.6%, −23.5%, and −31.5%, respectively; all p < 0.05) were comparable to those observed in the overall cohort, indicating consistent results in patients with high adherence (**Supplementary Figure 2**).

### Subgroup analysis

**Figure 2:**
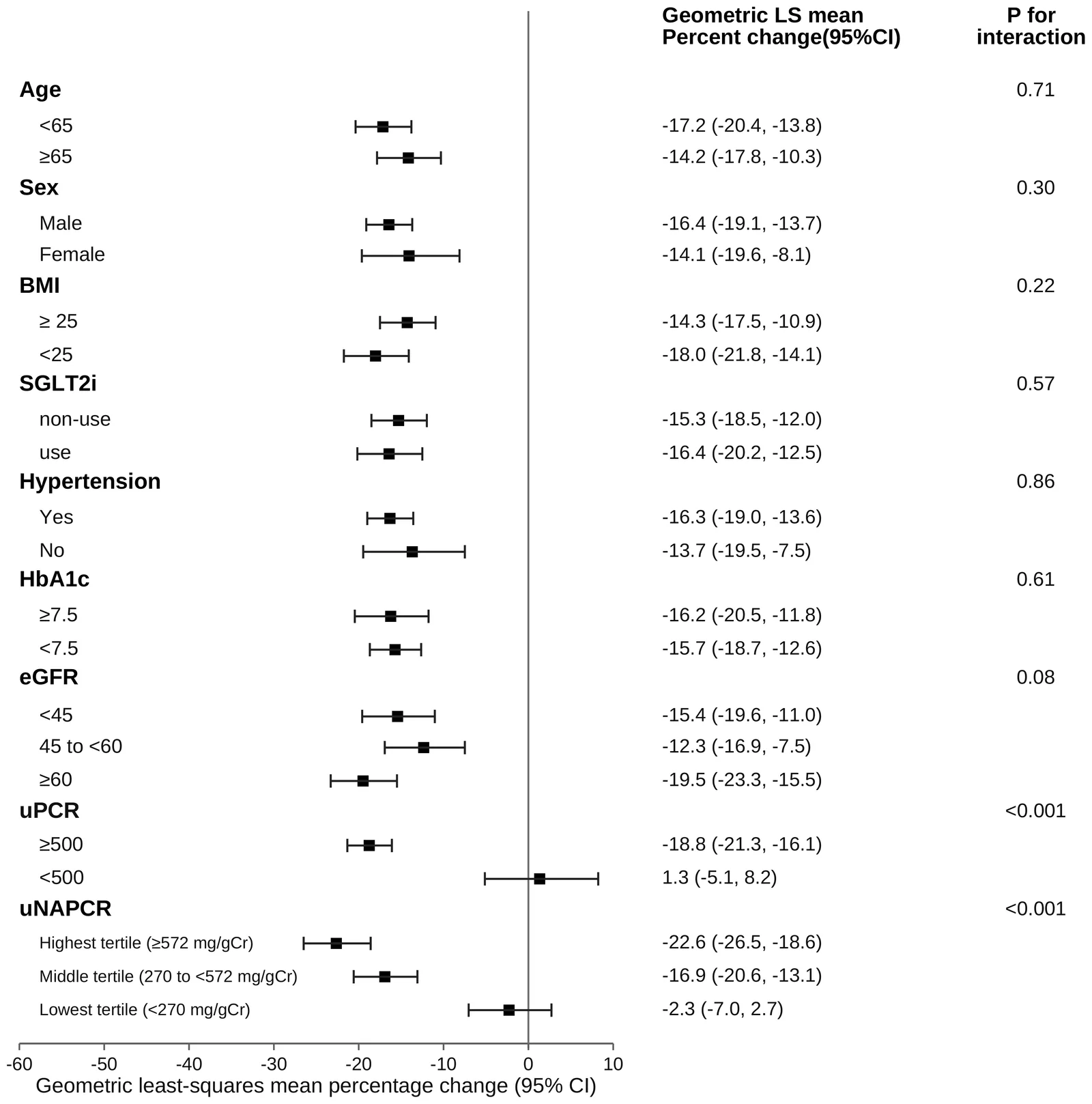
Subgroup forest plot of uNAPCR change at 6 months. Forest plot of geometric mean percentage change in uNAPCR from baseline to follow-up, estimated with a linear mixed-effects model adjusting for baseline age, sex, BMI, hypertension, HbA1c, SGLT2i use, eGFR and severity of proteinuria; stratification variables were excluded from covariate adjustment. For the uNAPCR subgroup interaction term, baseline proteinuria severity (uPCR category) was not included as an adjustment covariate. Error bars indicate 95% CIs. BMI, body mass index; HbA1c, hemoglobin A1c; SGLT2i, sodium-glucose cotransporter 2 inhibitor; eGFR, estimated glomerular filtration rate; uPCR, urine protein-to-creatinine ratio; uACR, urine albumin-to-creatinine ratio; uNAPCR, urine non-albumin protein-to-creatinine ratio.

**Figure 2** presents a forest plot of the geometric least-squares mean percentage change in uNAPCR from baseline to 6 months across prespecified subgroups. Reductions in uNAPCR were similar between participants aged <65 years and those ≥65 years, with changes of −17.2% (95% CI, −20.4 to −13.8) and −14.2% (95% CI, −17.8 to −10.3), respectively (p for interaction = 0.71). Comparable changes were also observed in males and females (−16.4% vs −14.1%; p for interaction = 0.30), and across subgroups defined by BMI, hypertension, HbA1c, and eGFR (all p for interaction > 0.05). Reductions in uNAPCR were likewise similar between patients without and with baseline SGLT2i use (−15.3% vs −16.4%; p for interaction = 0.57). In contrast, baseline proteinuria showed a clear difference: patients with uPCR ≥500 mg/gCr had a marked reduction in uNAPCR of −18.8% (95% CI, −21.3 to −16.1), whereas those with uPCR <500 mg/gCr showed little change (+1.3%, 95% CI, −5.1 to 8.2; p for interaction < 0.001). This gradient in the magnitude of change was further evident when stratifying by baseline uNAPCR levels (p for interaction < 0.001). Patients in the lowest tertile (tertile 1, <270 mg/gCr) showed a minimal, non-significant change of −2.3% (95% CI, −7.0 to 2.7). In contrast, a pronounced reduction of −16.9% (95% CI, −20.6 to −13.1) was observed in the middle tertile (tertile 2, 270 to <572 mg/gCr), and the largest reduction was seen in the highest tertile (tertile 3, ≥572 mg/gCr) at −22.6% (95% CI, −26.5 to −18.6). Overall, most subgroups showed a decrease in uNAPCR, and baseline urinary protein burden, reflected by both uPCR category and uNAPCR tertile, was the only factor for which the uNAPCR response differed significantly, with a clear reduction confined to patients with higher baseline proteinuria.

### Exploratory association between changes in uNAPCR and uNAGCR

**Figure 3:**
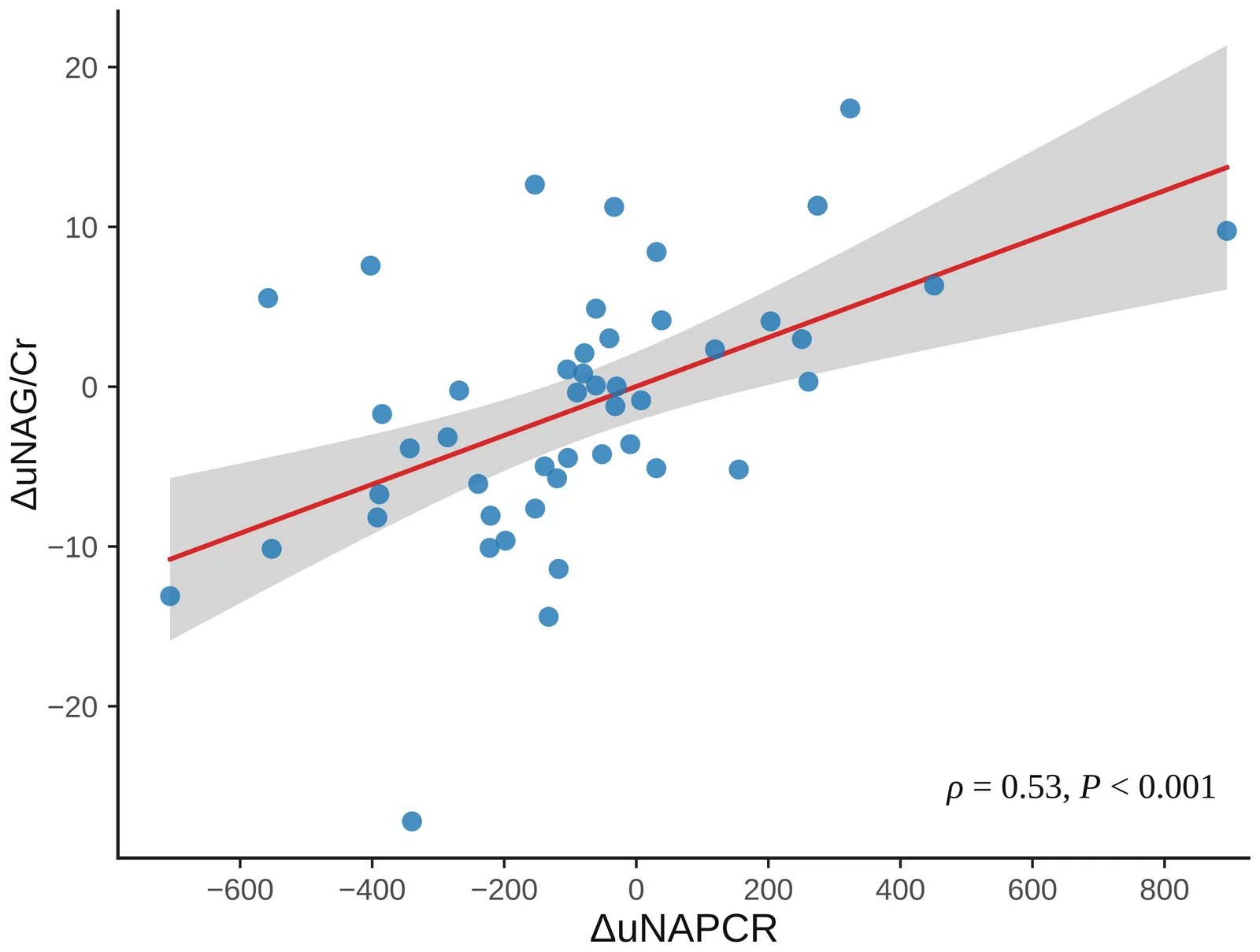
Association between changes in uNAPCR and uNAGCR. Each point represents one patient. The solid line represents the linear regression line; the shaded area represents the 95% CI. The two changes were positively correlated (Spearman’s ρ = 0.53; 95% CI, 0.29 to 0.70; p < 0.001). The association persisted after multivariable adjustment (Results). CI, confidence interval; uNAPCR, urine non-albumin protein-to-creatinine ratio; uNAG/Cr, urine N-acetyl-β-D-glucosaminidase-to-creatinine ratio; Δ, change from baseline.

Paired pre- and post-index uNAGCR measurements were available for 50 patients. Compared with patients without paired measurements, those with paired uNAGCR data had higher baseline eGFR (77.7 [47.4, 91.2] vs 53.0 [40.2, 68.0] mL/min/1.73 m²; p < 0.001), whereas age, sex, BMI, HbA1c, hypertension, baseline SGLT2i use, uPCR, uACR, and uNAPCR did not differ significantly (**Supplementary Table 1**). Within this subset, changes in uNAPCR (ΔuNAPCR) were positively associated with changes in uNAGCR (ΔuNAG/Cr), as assessed using Spearman’s rank correlation (ρ = 0.53; 95% CI, 0.29 to 0.70; p < 0.001) (**Figure 3**). This association remained significant after adjustment for age, sex, BMI, hypertension, HbA1c, SGLT2i use, eGFR, and baseline uNAPCR, with a partial ρ = 0.46 (p = 0.001). In a multivariable linear regression model including the same covariates, ΔuNAPCR was independently associated with ΔuNAG/Cr (β for ΔuNAPCR = 0.017; p = 0.002), indicating that greater reductions in uNAPCR were accompanied by larger decreases in uNAGCR. Calibration weighting eliminated the residual covariate imbalance (all |SMD| <0.001, from a pre-weighting maximum of 0.58 for baseline eGFR; effective sample size, 34.1 of 50). The calibration-weighted rank correlation was essentially unchanged (ρ = 0.52; 95% CI, 0.20 to 0.77; permutation p < 0.001) from the unweighted estimate (ρ = 0.53; 95% CI, 0.29 to 0.70) (**Supplementary Figure 3**).

### Longitudinal robustness analyses of uNAPCR change

In the analysis of pre- and post-treatment uNAPCR trends, restricted to patients with at least two pre-index uACR/uPCR measurements (233 patients), the pre-index uNAPCR slope was small and not statistically significant (−2.1% per 6 months; 95% CI, −6.6 to 2.6; p = 0.38), whereas the post-index slope showed a significant decline (−20.4% per 6 months; 95% CI, −24.8 to −15.7; p < 0.001). The post-index slope was significantly lower than the pre-index slope (slope difference, −18.7% per 6 months; 95% CI, −25.1 to −11.7; p < 0.001) (**Supplementary Figure 4**).

### Safety outcomes for hyperkalemia

Among the 518 patients eligible for the safety analysis (all finerenone recipients irrespective of the ≥90-day criterion), 106 patients (20.5%) experienced at least one episode of hyperkalemia, defined as serum potassium >5.5 mmol/L, and 5 patients (1.0%) experienced severe hyperkalemia (>6.5 mmol/L). When stratified by baseline eGFR, the incidence of hyperkalemia was highest in patients with eGFR <45 (60 of 181, 33.1%), followed by those with eGFR 45 to <60 (31 of 143, 21.7%) and eGFR ≥60 (15 of 194, 7.7%). Severe hyperkalemia occurred in 2 patients (1.1%) with eGFR <45 and in 3 patients (2.1%) with eGFR 45 to <60, whereas no cases were observed in patients with eGFR ≥60 (**Table 2**).

**Table 2:** Safety outcomes for hyperkalemia.

Characteristic		eGFR category		
	Overall	<45	45 to <60	≥60
Number of patients^*^	518	181	143	194
Follow-up period^†^	6.5 [3.3, 10.3]	6.5 [3.3, 11.2]	6.7 [3.2, 9.7]	6.5 [3.7, 10.3]
Hyperkalemia	106 (20.5)	60 (33.1)	31 (21.7)	15 (7.7)
Severe hyperkalemia	5 (1.0)	2 (1.1)	3 (2.1)	0 (0.0)

Number of patients with one or more episodes of hyperkalemia during the follow-up period, categorized by eGFR category. Data are n (%), unless otherwise indicated. Percentages for eGFR categories are calculated within each stratum; overall percentages are based on the total population. Hyperkalemia was defined as serum potassium >5.5 mmol/L. Severe hyperkalemia was defined as serum potassium >6.5 mmol/L.

* All patients with any finerenone prescription who had available baseline eGFR and at least one follow-up serum potassium measurement.

† Follow-up duration is expressed in months as median [Q1, Q3].

## Discussion

Finerenone, a selective nonsteroidal MR antagonist, has been shown to improve renal and cardiovascular outcomes and reduce albuminuria in patients with T2D and CKD (17, 18). However, albuminuria primarily reflects glomerular injury, whereas the non-albumin fraction of proteinuria (NAP) is enriched for tubule-derived proteins, particularly low-molecular-weight proteins associated with tubular injury, and has been used as a marker of tubular damage (4, 23). We hypothesized that the anti-inflammatory and anti-fibrotic properties of finerenone would contribute to tubular protection, as reflected by NAP reduction beyond its established albuminuria-lowering effect.

In our real-world cohort, which included patients with moderately reduced kidney function (median eGFR 53.7 mL/min/1.73 m²), we observed four key findings. First, finerenone treatment was associated with an 18.8% reduction in uNAPCR from baseline at 6 months among patients with severe proteinuria (uPCR ≥ 500 mg/gCr). Second, declines in uNAPCR were consistent across age, sex, BMI, hypertension, HbA1c, eGFR, and SGLT2i use. Third, changes in uNAPCR were significantly correlated with changes in uNAG, a well-established urinary marker of tubular injury, even after adjustment for clinical covariates. Finally, albuminuria decreased by 30.3% after finerenone initiation, to an extent comparable to that reported in previous randomized controlled trials (17, 19), thereby supporting the external validity of our retrospective findings.

We selected uNAPCR as our primary outcome for both its clinical feasibility and established prognostic relevance. Although biomarkers such as kidney injury molecule-1 (KIM-1) and neutrophil gelatinase-associated lipocalin (NGAL) are highly specific for tubular injury (24), they require specialized immunoassays, and their clinical standardization and widespread availability remain limited. In contrast, uNAPCR can be readily calculated from two routine tests (uPCR and uACR), which are often obtained from the same spot-urine specimen. This enables broad implementation in real-world settings without adding the burden of additional specialized assays. Beyond these practical considerations, we also sought to emphasize the scientific rationale for adopting uNAPCR as our primary tubular biomarker. Current guidelines classify CKD risk using eGFR and albuminuria, reflecting an albuminuria-centered approach to risk stratification (1, 22). Large randomized trials of finerenone, including FIDELIO-DKD and FIGARO-DKD, have likewise focused on albuminuria reduction, demonstrating that finerenone reduces albuminuria (17, 18, 25) while mitigating cardiorenal events in patients with T2D (17, 18). However, non-albumin proteinuria is given far less emphasis despite its prognostic significance. Previous studies have specifically defined uNAPCR as uPCR − uACR and have shown that elevated uNAPCR is associated with increased tubular injury markers, mortality and CKD progression in T2D, independently of albuminuria (4, 6, 7). Furthermore, a Korean cohort study reported that approximately 15% of T2D patients with normoalbuminuria had proteinuria (5).

Our data suggest that finerenone treatment is associated with reductions in both albuminuria and uNAPCR, the latter of which is enriched for non-albumin proteins including tubular proteins. This observation is consistent with mechanistic evidence that overactivation of the MR drives oxidative stress, inflammation, and fibrosis in the tubulointerstitium (14, 15, 26). Finerenone attenuates these pathways by selectively modulating MR cofactors and thereby suppressing proinflammatory and profibrotic gene expression (11–13). Consistent with this mechanistic framework, the magnitude of change in uNAPCR was significantly correlated with changes in uNAGCR, a well-established urinary marker of tubular injury (Spearman’s ρ = 0.53; p < 0.001). This association remained significant after adjustment for multiple clinical covariates (partial ρ = 0.46; p = 0.001), supporting the interpretation that the observed uNAPCR reduction may reflect improvement in tubular injury-related processes. While the observed decline in uACR mirrors declines reported in previous trials (17, 19), the reduction in uNAPCR may reflect changes in the tubulointerstitial compartment, a response not captured in prior finerenone studies.

This correlation, however, was derived from the subset in whom uNAG was measured at the discretion of the treating physician. These patients had substantially better baseline kidney function than the remainder of the cohort, but calibration weighting to the full cohort left the correlation essentially unchanged (**Supplementary Figure 3**), indicating that the observed baseline imbalance was unlikely to have materially accounted for the association. However, weighting can balance only measured characteristics; confirmation will therefore require prospective studies with protocol-driven measurement of specific tubular injury biomarkers in all participants.

The greater apparent reduction at higher levels of baseline proteinuria is biologically plausible, as a higher urinary protein load may indicate more extensive tubulointerstitial injury. In clinical practice, measuring uNAPCR in conjunction with uACR may refine the phenotyping of DKD by capturing tubulointerstitial injury that albuminuria alone does not index (4, 6, 23). Within this framework, baseline proteinuria severity emerges as a candidate phenotype for identifying patients in whom uNAPCR is most responsive to finerenone, although this requires prospective validation. Because uNAPCR can be derived from two routinely available laboratory measurements (uPCR and uACR), a uNAPCR-guided approach to finerenone therapy could be evaluated even where dedicated tubular biomarker assays are not performed.

Our analyses showed no evidence of a differential change according to baseline SGLT2i use (p for interaction = 0.57). This finding aligns with an exploratory analysis of predefined subgroups in FIDELIO-DKD, which showed that the magnitude of albuminuria reduction with finerenone did not differ significantly according to baseline SGLT2i therapy (27). Across predefined subgroups based on age, sex, BMI, hypertension, HbA1c, and eGFR strata, finerenone was associated with consistent reductions in uNAPCR with no reversal of the direction of change.

In our study, laboratory-defined hyperkalemia (serum potassium > 5.5 mmol/L) occurred in 20.5% of patients, and severe hyperkalemia (> 6.5 mmol/L) in 1.0%, with events predominantly clustered in those with eGFR <60 mL/min/1.73 m². The overall frequency of hyperkalemia is thus broadly comparable to that observed in FIDELIO-DKD for similar potassium thresholds (17). The observed frequency was higher than that reported in real-world studies using event-based definitions or adverse-event reporting, likely because our analysis captured any laboratory value above 5.5 mmol/L during follow-up (28, 29). Importantly, the steep gradient in hyperkalemia risk across eGFR categories in our cohort reproduces patterns seen in prior finerenone analyses, underscoring the need for closer potassium monitoring in patients with more advanced CKD.

This study has several limitations that warrant consideration. First, this study was a single-arm before-after analysis without a concurrent comparator, so our findings establish association rather than causation, and residual confounding, co-interventions, secular trends, and regression to the mean cannot be excluded. However, because regression to the mean reflects spontaneous return from a randomly elevated baseline, we examined the pre-treatment trajectory using multiple pre-index measurements per patient: uNAPCR was stable before finerenone and declined steeply only after initiation (**Supplementary Figure 4**), indicating that the elevated baseline was sustained rather than transiently high and that the decline did not represent the continuation of a pre-existing trend. This pattern, together with the uACR decline consistent with that reported in previous randomized trials, makes regression to the mean alone an unlikely explanation. Second, uNAPCR is a derived quantity rather than a directly measured analyte: it is obtained as the difference between two separately calibrated assays, uPCR and uACR, which also differ in analyte specificity, and therefore carries the measurement error of both. We limited the variability under our control by requiring that uPCR and uACR originate from the same spot-urine specimen analyzed on the same platform, and by converting all values to common units before subtraction. No derived uNAPCR value fell to zero or below—the principal concern when a quantity is defined by subtraction—yet residual misclassification remains possible, particularly near the lower range of proteinuria. Urinary protein excretion is also subject to within-individual biological variation, so our reliance on single spot specimens adds random, non-directional noise to the estimated changes. Third, selection and information biases are possible because only patients who had uPCR and uACR measured concurrently were included, and exposure and adherence were derived from EMR. However, in the subgroup with high adherence (PDC ≥80%), the estimated 6-month reductions in uNAPCR, uPCR, and uACR were comparable to those in the overall cohort (**Supplementary Figure 2**), indicating that our estimates are not materially driven by heterogeneity in adherence. Fourth, it remains uncertain whether the observed reduction in uNAPCR translates into meaningful clinical outcomes; available follow-up was insufficient to characterize long-term eGFR trajectories, and the threshold of uNAPCR reduction required for clinical benefit remains to be determined in future studies. Fifth, this single-center Korean cohort may limit the generalizability of our findings to other ethnicities.

## Conclusion

In this real-world cohort of patients with T2D, finerenone treatment was associated with significant reductions in NAP among those with higher baseline proteinuria, accompanied in an exploratory subset by a parallel decline in a urinary marker of tubular injury. These findings are hypothesis-generating and suggest that the response to finerenone may extend to tubulointerstitial injury-related processes beyond albuminuria, a possibility that our study design cannot confirm. Because uNAPCR is derived from two routinely available urine tests, it provides an accessible way to look beyond albuminuria toward the tubulointerstitial compartment. We therefore present uNAPCR as a candidate biomarker of treatment response to finerenone, which warrants evaluation in prospective, multicenter studies with clinical endpoints.

## Data Availability

The data analyzed in this study is subject to the following licenses/restrictions: Aggregate datasets can be obtained from the corresponding author upon reasonable request. Requests to access these datasets should be directed to Byung-Wan Lee, bwanlee@yuhs.ac.
