# Supplementary material for: Non-albumin proteinuria as a candidate biomarker of treatment response to finerenone in diabetic kidney disease: a real-world cohort study
Source: Front Endocrinol (Lausanne). 2026 Sep 8;17:1918192. doi: 10.3389/fendo.2026.1918192 (PMC13593432; doi:10.3389/fendo.2026.1918192)
Supplement: Supplementary file 1 [file DataSheet1.docx]

**Supplementary Table S1 | Baseline characteristics according to paired uNAGCR availability.**

| **Characteristic** | **Paired uNAGCR available**  **(n = 50)** | **Paired uNAGCR unavailable**  **(n = 427)** | **P value** |
| --- | --- | --- | --- |
| Age, years | 66.1 (10.1) | 66.4 (12.6) | 0.89 |
| Male, n (%) | 36 (72.0) | 304 (71.2) | 1.00 |
| BMI, kg/m² | 25.9 [24.2, 28.6] | 25.7 [23.4, 28.1] | 0.33 |
| HbA1c, % | 7.5 (1.5) | 7.4 (1.4) | 0.61 |
| Hypertension, n (%) | 44 (88.0) | 357 (83.6) | 0.54 |
| Baseline SGLT2 inhibitor use, n (%) | 17 (34.0) | 194 (45.4) | 0.13 |
| eGFR, mL/min/1.73 m² | 77.7 [47.4, 91.2] | 53.0 [40.2, 68.0] | **<0.001** |
| eGFR category, n (%) |  |  | **<0.001** |
| ≥60 mL/min/1.73 m² | 32 (64.0) | 157 (36.8) |  |
| 45 to <60 mL/min/1.73 m² | 7 (14.0) | 124 (29.0) |  |
| <45 mL/min/1.73 m² | 11 (22.0) | 146 (34.2) |  |
| uPCR, mg/gCr | 1,065 [642, 1,595] | 1,180 [565, 2,350] | 0.69 |
| uACR, mg/gCr | 655 [382, 1,135] | 720 [316, 1,667] | 0.72 |
| uNAPCR, mg/gCr | 392 [237, 538] | 417 [221, 709] | 0.61 |

Data are expressed as mean (SD) for age and HbA1c, median [interquartile range] for other continuous variables, and number (%) for categorical variables.

P values were calculated using the two-sided Welch t test for age and HbA1c, the two-sided Mann-Whitney U test for other continuous variables, and the two-sided Fisher exact test for binary variables. The eGFR-category distribution used the Pearson chi-square test. P value <0.05 is shown in bold.

BMI, body mass index; HbA1c, hemoglobin A1c; SGLT2, sodium-glucose cotransporter 2; eGFR, estimated glomerular filtration rate; uPCR, urine protein-to-creatinine ratio; uACR, urine albumin-to-creatinine ratio; uNAPCR, urine non-albumin protein-to-creatinine ratio.

**Supplementary Figure S1 | Flow chart of patient inclusion/exclusion and data analysis.** The dashed box denotes the safety analysis population. eGFR, estimated glomerular filtration rate; PDC, proportion of days covered; uACR, urine albumin-to-creatinine ratio; uNAG, urine N-acetyl-β-D-glucosaminidase; uNAGCR, urine N-acetyl-β-D-glucosaminidase-to-creatinine ratio; uNAPCR, urine non-albumin protein-to-creatinine ratio; uPCR, urine protein-to-creatinine ratio; Δ, change from baseline.

**Supplementary Figure S2 | Geometric Mean Percentage Changes in uPCR/uACR/uNAPCR after 6 Months of Finerenone Treatment in the High-Adherence Group (PDC ≥80%).** Analyses were restricted to patients with finerenone PDC ≥80% during the exposure. A linear mixed-effects model was used to estimate percent change from baseline to 6 months, adjusting for age, sex, BMI, hypertension, HbA1c, SGLT2i use, eGFR and severity of proteinuria. The error bars denote 95% confidence intervals. * p < 0.05. uACR, urine albumin-to-creatinine ratio; uNAPCR, urine non-albumin protein-to-creatinine ratio; uPCR, urine protein-to-creatinine ratio; PDC, proportion of days covered.

**Supplementary Figure S3 | Calibration weighting and association between changes in uNAPCR and uNAGCR.** (A) Absolute standardized mean differences between the 50-patient association subset and the full analytic cohort before and after calibration weighting. Calibration weights were estimated by entropy balancing for age, sex, baseline BMI, hypertension, HbA1c, baseline SGLT2 inhibitor use, eGFR, and log-transformed baseline uNAPCR; only variables included in the calibration constraints are shown. The vertical dashed line indicates an absolute standardized mean difference of 0.10. (B) Unweighted Spearman's correlation and calibration-weighted rank correlation between changes in uNAPCR and uNAGCR, shown with 95% confidence intervals. Calibration weights ranged from 0.17 to 2.56 and yielded an effective sample size of 34.1. The p value for the calibration-weighted correlation was obtained using a permutation test. BMI, body mass index; CI, confidence interval; eGFR, estimated glomerular filtration rate; HbA1c, hemoglobin A1c; SMD, standardized mean difference; uNAGCR, urine N-acetyl-β-D-glucosaminidase-to-creatinine ratio; uNAPCR, urine non-albumin protein-to-creatinine ratio.

**Supplementary Figure S4 | Pre- versus post-index trajectory of uNAPCR from a continuous linear-spline mixed-effects model.** Two linear time terms with a single knot at the index date allowed separate pre-index and post-index slopes; the model used all available longitudinal same-specimen measurements (restricted to patients with at least two pre-index measurements). Before initiation, uNAPCR showed no significant change (grey segment; pre-index slope −2.1% per 6 months; 95% CI −6.6 to 2.6; p = 0.38), whereas a marked decline emerged afterward (red segment; post-index slope −20.4% per 6 months; 95% CI −24.8 to −15.7; p < 0.001), with a significant between-slope difference (slope difference −18.7% per 6 months; 95% CI −25.1 to −11.7; p < 0.001). Shaded bands represent the 95% confidence intervals implied by the slope estimates, anchored at the index value; the vertical dashed line marks finerenone initiation. CI, confidence interval; uNAPCR, urine non-albumin protein-to-creatinine ratio.

**Supplementary Figure S5 | Starting-to-final finerenone dose transitions in the cohort.** The starting dose is the daily dose of the first prescription, and the final dose is the daily dose of the last prescription within the episode. Node heights are proportional to the number of patients at each dose (5, 10, or 20 mg/day) at initiation (left) and at the final prescription (right); ribbon widths are proportional to the number of patients following each starting-to-final transition. Overall, 366 patients (76.7%) remained on their starting dose, 94 (19.7%) were up-titrated, and 17 (3.6%) were down-titrated.
